# Clinical Significance of PD‐L1 and HLA Expression in Esophageal Squamous Cell Carcinoma in Response to Immunotherapy

**DOI:** 10.1002/ags3.70227

**Published:** 2026-05-08

**Authors:** Kosuke Kanemitsu, Yoshiyuki Tagayasu, Yoshihiro Komohara, Cheng Pan, Rin Yamada, Yukio Fujiwara, Kazuto Harada, Keisuke Kosumi, Yoshifumi Baba, Masaaki Iwatsuki

**Affiliations:** ^1^ Department of Cell Pathology, Graduate School of Medical Sciences Kumamoto University Kumamoto Japan; ^2^ Department of Gastroenterological Surgery, Graduate School of Medical Sciences Kumamoto University Kumamoto Japan; ^3^ Center for Metabolic Regulation of Healthy Aging Kumamoto University Kumamoto Japan; ^4^ Department of Gastrointestinal Surgery Graduate School of Medicine the University of Tokyo Tokyo Japan

**Keywords:** CD169, esophagus, HLA, macrophage, PD‐L1, squamous cell carcinoma

## Abstract

Despite recent advances in multimodal therapy, esophageal squamous cell carcinoma (ESCC) remains a highly lethal malignancy. Although immune checkpoint inhibitors (ICIs) have improved clinical outcomes, predictive biomarkers are urgently needed. This study aimed to investigate immune‐related features in tumor cells and the tumor microenvironment in pathological specimens from 45 patients with ESCC treated with ICIs for postoperative recurrence after curative resection. Immunohistochemistry and quantitative image analysis of CD3, CD8, FoxP3, CD163, PD‐L1, HLA‐A/B/C, and HLA‐DR were conducted. High programmed death‐ligand 1 (PD‐L1) expression in tumor‐associated macrophages (TAMs) and tumor cells was significantly associated with a favorable response. Infiltration of T cells, regulatory T cells, and TAMs was not associated with the clinical response, but increased T‐cell infiltration was closely associated with high PD‐L1 expression in TAMs. Downregulation of HLA‐A/B/C and ectopic HLA‐DR expression was seen in responders. High PD‐L1 expression in TAMs was closely related to increased T‐cell infiltration, and single‐cell RNA‐sequence analysis suggested a significant correlation with T‐cell proliferation. Collectively, these findings indicate that not only PD‐L1, but also HLA expression is useful for predicting the effectiveness of ICI therapy in patients with ESCC.

## Graphical abstract

This study evaluated immune‐related biomarkers in esophageal squamous cell carcinoma treated with immune checkpoint inhibitors after postoperative recurrence. PD‐L1 expression in tumor‐associated macrophages and tumor cells, together with HLA class I downregulation and HLA‐DR expression, were associated with favorable responses to immunotherapy. These findings suggest that PD‐L1 and HLA expression patterns may serve as useful biomarkers for predicting immunotherapy efficacy in ESCC.

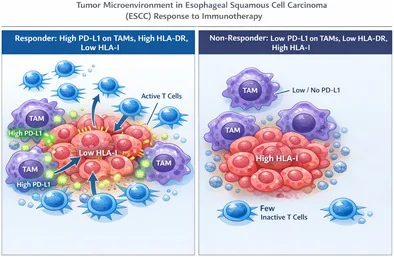

## 1 Introduction

Esophageal squamous cell carcinoma (ESCC) is among the most aggressive malignancies of the gastrointestinal tract and continues to impose a substantial global oncologic burden. Its incidence is particularly high in East Asia, where ESCC remains the predominant histological subtype of esophageal cancer [1]. Despite advances in multimodal treatment approaches—including surgery, chemotherapy, and radiotherapy—long‐term outcomes for patients with advanced or recurrent ESCC remain unsatisfactory [2].

The advent of immune checkpoint inhibitors (ICIs), particularly antibodies targeting the programmed cell death 1 (PD‐1)/programmed death‐ligand 1 (PD‐L1) axis, has transformed the therapeutic landscape across multiple cancers. In ESCC, landmark trials such as KEYNOTE‐590 have demonstrated that the addition of pembrolizumab to chemotherapy significantly improves overall survival compared with chemotherapy alone, especially in patients with higher PD‐L1 expression [3]. Likewise, CheckMate‐648 reported survival benefits with nivolumab‐based regimens in ESCC [4]. These findings have established ICIs as standard components of systemic therapy for advanced ESCC. Nevertheless, clinical benefits are still confined to a subset of patients, and durable responses are not universal. Therefore, the identification of reliable predictive biomarkers is essential to refine patient selection and maximize therapeutic efficacy.

The responsiveness to ICIs is largely shaped by the immunological landscape of the tumor microenvironment (TME). Tumors with abundant immune infiltration—so‐called “hot tumors”—are more likely to respond to ICIs, whereas “cold tumors,” characterized by sparse immune infiltration, are typically resistant [5]. Among tumor‐infiltrating lymphocytes (TILs), CD3+ and CD8+ T cells have been extensively studied across various malignancies and are generally associated with improved survival and enhanced responses to ICIs [6, 7]. However, in ESCC, the predictive value of these T‐cell subsets remains inconsistent and incompletely defined. By contrast, immunosuppressive immune cell populations such as FoxP3+ regulatory T cells (Tregs) and CD163+ tumor‐associated macrophages (TAMs) have been shown to promote tumor immune evasion and are often linked to a poor prognosis [8, 9].

Beyond PD‐L1, additional immune regulatory mechanisms also shape responses to ICIs. The integrity of the antigen‐presentation machinery is essential for effective antitumor immunity. HLA class I molecules (HLA‐A, HLA‐B, and HLA‐C) present tumor antigens to cytotoxic CD8+ T cells, whereas HLA class II molecules such as HLA‐DR mediate antigen presentation to CD4+ T cells. Downregulation of HLA class I has been reported across multiple cancers and is often associated with immune escape and resistance to immunotherapy [10, 11]. In several tumor types, including melanoma and lymphoma, the ectopic expression of HLA‐DR in tumor cells has been shown to predict favorable responses to ICI therapy [12, 13]. However, to our knowledge, no studies to date have examined the relationship between HLA expression and response to ICI therapy in ESCC.

Taken together, these observations underscore the need for a comprehensive evaluation of immune biomarkers that extends beyond PD‐L1 expression and immune cell infiltration alone. Therefore, in this study, we examined tumor specimens from patients with ESCC treated with ICIs for postoperative recurrence or who underwent conversion surgery after initially unresectable disease. Using immunohistochemistry and quantitative digital pathology, we analyzed the expression of HLA molecules together with CD3, CD8, FoxP3, CD163, and PD‐L1.

## 2 Material and Methods

### 2.1 Patients and Tissue Samples

Among the 48 patients who received ICI therapy, 45 were treated for postoperative recurrence after curative resection, whereas three underwent conversion surgery following systemic therapy for initially unresectable disease. To ensure consistency in specimen timing and treatment context, the primary analysis was restricted to the 45 cases of postoperative recurrence. All patients included in the primary analysis initially underwent curative surgical resection. Recurrence was confirmed radiologically during follow‐up, and ICI therapy was initiated after confirmation of postoperative recurrence. The interval between surgery and recurrence varied according to the individual clinical course.

Patients received ICI‐based regimens according to the clinical indication at the time of recurrence, including nivolumab monotherapy (*n* = 25), nivolumab plus ipilimumab combination therapy (*n* = 9), and fluoropyrimidine plus cisplatin combined with pembrolizumab (*n* = 11). The line of therapy varied depending on prior systemic treatment history. Treatment response was evaluated according to the Response Evaluation Criteria in Solid Tumors, version 1.1. The best overall response during ICI therapy was recorded. Patients who achieved a complete response or partial response were classified as responders, whereas those with stable disease or progressive disease were classified as nonresponders. In the revised analysis, hematologic parameters were defined as baseline values obtained within 7 days prior to initiation of ICI therapy.

### 2.2 Immunohistochemistry

Formalin‐fixed, paraffin‐embedded tissue blocks were sectioned at 3 μm and subjected to immunohistochemical staining. The following primary antibodies were used: anti‐CD3, anti‐CD8, anti‐FoxP3, anti‐CD163, anti‐PD‐L1, anti‐HLA‐A/B/C (class I), and anti‐HLA‐DR (class II). Detection was performed using horseradish peroxidase‐conjugated secondary antibodies and visualized with the DAB chromogen system (Nichirei, Tokyo, Japan). Cell counting of CD3‐, CD8‐, Foxp3‐, or CD163‐positive cells in the tumor nest and stroma was performed using HALO version 3.6.4134 (Indica Labs, Albuquerque, NM, USA). HLA‐A/B/C expression was evaluated based on membranous staining intensity in tumor cells, using adjacent non‐neoplastic epithelial or stromal cells as internal controls. Downregulation was defined as complete loss or markedly reduced membranous staining compared with internal controls. HLA‐DR expression was considered positive (ectopic expression) when ≥ 10% of tumor cells exhibited definite membranous staining. All slides were independently evaluated by two board‐certified pathologists blinded to clinical information, and any discrepancies were resolved by consensus.

### 2.3 Analysis of Single‐Cell RNA‐Sequence (Sc‐RNA‐Seq) Data

The CD45+ cell sc‐RNA‐seq data of 60 ESCC cases were obtained from an open data set (GSE160269). Analysis was conducted using the R package *Seurat*. Briefly, the counts were normalized and 2000 highly variated genes were identified for principal component analysis. The normalized counts were scaled and the batch effects of each case were reduced using the R package *harmony*. The top 50 components were used for uniform manifold approximation and projection (UMAP) dimension reduction and clustering. In total, 17 clusters (0–16) were identified with a resolution of 0.4. In addition, AIF1‐high expressing clusters 4, 10, 14, 15, and 16 were annotated as myeloid‐lineage cells, and CD3D‐ and CD3E‐high expressing clusters 0, 1, 2, 3, 8, and 9 were annotated as T cells. The myeloid‐lineage cells were further clustered into 12 clusters (0–11), and TAMs (APOE+, MSR[CD204]+, CD68, and CD163+ clusters 0, 1, 4, and 8) were extracted for the examination of CD274 (PD‐L1) expression. T cells were also further clustered into 27 clusters (0–26). CD8A‐ and CD8B‐high expressing 0, 4, 5, 7, 8, 9, 10, 11, 13, 14, 17, 22, and 24 were annotated as CD8 T cells, whereas CD4‐high expressing 1, 2, 3, 6, 11, 12, 15, 16, 18, 19, 20, 21, and 23 were annotated as CD4 T cells. Among CD8 and CD4 T cells, MKI67‐positive clusters 9 and 11 were considered proliferating CD8 T cells, and clusters 18 and 19 were considered proliferating CD4 T cells.

### 2.4 Statistical Analysis

The prognostic nutritional index (PNI) was calculated using the following formula: PNI = 10 × serum albumin (g/dL) + 0.005 × total peripheral lymphocyte count (/mm^3^). All statistical analyses were conducted using GraphPad Prism (GraphPad Software, San Diego, CA, USA). Differences between groups were assessed using the Mann–Whitney *U* test and the Kruskal–Wallis test or chi‐square test, as appropriate. P‐values < 0.05 were considered statistically significant. Progression‐free survival (PFS) was defined as the interval from initiation of ICI therapy to disease progression or death, with censoring at last follow‐up.

## 3 Results

### 3.1 Patient Characteristics

A total of 45 patients with recurrent or initially unresectable ESCC who had received ICIs were included in this study. Based on clinical outcomes, 12 patients were classified as responders and 33 as nonresponders. The participants' baseline clinical characteristics are summarized in Table 1. The median age of the cohort was 66 years (interquartile range, 62–71), and most patients were male (75.5%). No significant differences in sex, age, or performance status were observed between responders and nonresponders. However, body mass index was significantly higher in responders than in nonresponders. Regarding baseline hematologic parameters prior to the initiation of ICI therapy, responders had significantly higher hemoglobin levels and higher PNI values than nonresponders. Although responders tended to have lower neutrophil percentages and C‐reactive protein (CRP) levels, these differences did not reach statistical significance. Serum albumin levels also tended to be higher in responders, but the difference was not statistically significant. Squamous cell carcinoma antigen levels, platelet counts, and eosinophil percentages did not differ significantly between the two groups.

**TABLE 1:** Clinical characteristics compared between responders and nonresponders.

Characteristic median (IQR)	All patients (*n* = 45)	Efficacy of immunotherapy		*p*‐value
		Responders (*n* = 12)	Nonresponders (*n* = 33)	
Sex (men)	34 (75.5%)	10 (83.3%)	24 (72.7%)	0.45
Age (years)	66.0 (62–71)	67 (63.3–76)	65 (60.5–70.5)	0.24
Body mass index (kg/m^2^)	19.4 (16.4–20.8)	20.8 (19.4–23.9)	18.3 (16.1–20.2)	**0.02**
Performance Status				0.16
1	4 (8.9%)	0 (0%)	4 (12.1%)	
2	1 (2.2%)	0 (0%)	1 (3.0%)	
Before ICI treatment WBC (×10^3^/μL)	5.3 (4.0–6.5)	5.6 (3.83–6.73)	5.2 (4.1–6.5)	0.86
Before ICI treatment Neut (%)	66.7 (58.5–71.2)	60.1 (57.2–72.1)	67.9 (61.8–71.1)	0.19
Before ICI treatment Eosin (%)	1.9 (1.05–4.3)	2.1 (1.23–4.48)	1.8 (0.90–4.25)	0.67
Before ICI treatment Hb (g/dL)	10.8 (10.1–12)	12.4 (10.9–13.3)	10.5 (9.7–11.8)	**0.005**
Before ICI treatment Plt (×10^4^/μL)	26.4 (20.4–31.1)	26.8 (20.7–29.9)	24.3 (20.0–32.2)	0.50
Before ICI treatment Alb (g/dL)	3.7 (3.40–3.95)	3.85 (3.5–4.0)	3.6 (3.3–3.8)	0.10
Before ICI treatment CRP (mg/dL)	0.23 (0.09–0.92)	0.18 (0.07–0.32)	0.23 (0.12–1.65)	0.07
Before ICI treatment SCC (ng/mL)	1.80 (1.20–3.05)	1.85 (1.23–3.0)	1.80 (1.20–3.10)	0.15
Before ICI treatment PNI	42.6 (39.3–45.8)	44.1 (41.9–48.1)	41.0 (38.8–44.8)	**0.04**

*Note:* Categorical variables are presented as proportions. Non‐normally distributed variables are reported as medians with interquartile ranges. Categorical data were compared using the chi‐square test or Fisher’s exact test. Non‐normally distributed data were compared using the Mann–Whitney *U* test. Abbreviations: SCC, squamous cell carcinoma antigen; IQR, interquartile range; PNI, prognostic nutritional index.

### 3.2 PD‐L1 Expression in Macrophages Predicted a Favorable Response to ICI Therapy

**FIGURE 1:**
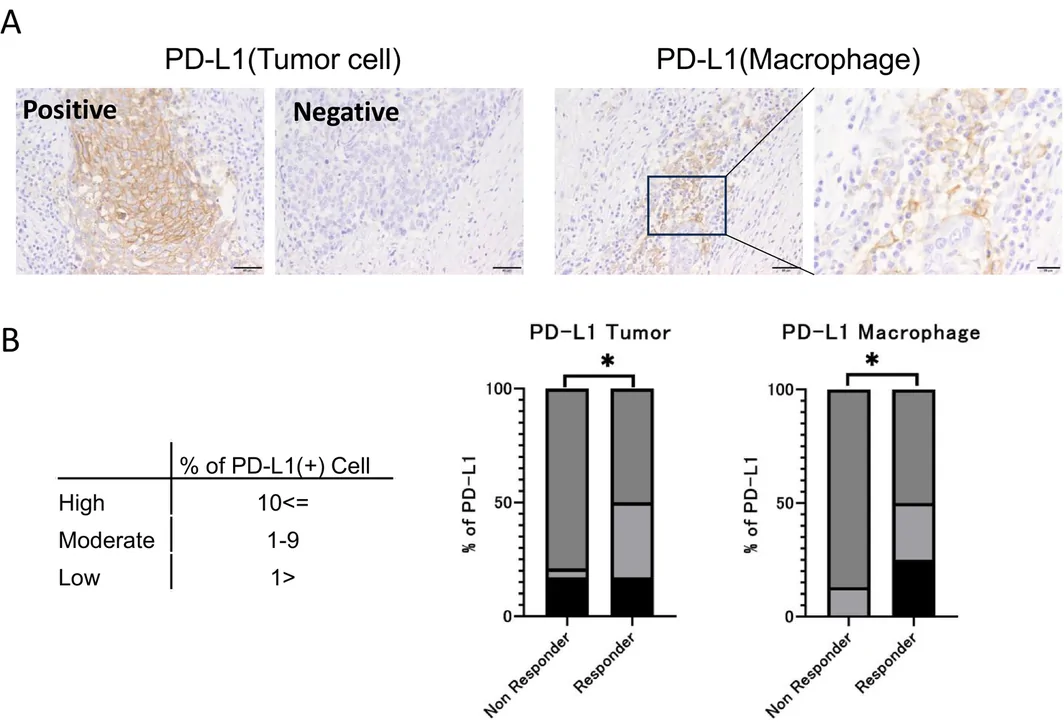
PD‐L1 expression in tumor cells and macrophages. (A) PD‐L1 expression was assessed in both tumor cells and macrophages. (B) Stacked bar graphs show the proportions of cases with low (< 1%), moderate (1%–9%), and high (≥ 10%) PD‐L1 expression in tumor cells and tumor‐associated macrophages. Dark gray, light gray, and moderate gray indicate high, moderate, and low PD‐L1 expression, respectively. *p < 0.001.

As previously described [14], PD‐L1 expression was observed in both cancer cells and TAMs and classified as follows into three groups according to the proportion of PD‐L1–positive cells: low (< 1%), intermediate (1%–10%), and high (> 10%) (Figure 1A). In cancer cells, the numbers of cases with low, intermediate, and high PD‐L1 expression were 31 (68.8%), 7 (15.6%), and 7 (15.6%), respectively. In TAMs, the corresponding numbers were 3 (6.7%), 12 (26.7%), and 30 (66.6%). Statistical analysis showed that PD‐L1 expression in TAMs and tumor cells was significantly higher in responders than in nonresponders (Figure 1B). Table 2 summarizes the relationship between macrophage PD‐L1 status and systemic inflammatory or nutritional markers. Patients with high or intermediate PD‐L1 expression in macrophages had lower neutrophil percentages. Other variables, including hemoglobin, albumin, CRP, and PNI, did not differ significantly across the PD‐L1 subgroups.

**TABLE 2:** Clinical characteristics according to PD‐L1 expression in macrophages.

Characteristic median (IQR)	All patients (*n* = 45)	PD‐L1 expression in macrophages			*p*‐value
		High (*n* = 3)	Moderate (*n* = 12)	Low (*n* = 30)	
Before ICI treatment WBC (×10^3^/μL)	5.3 (4.0–6.5)	5.6 (5.30–5.60)	4.7 (3.55–6.28)	5.1 (4.00–6.53)	0.68
Before ICI treatment Neut (%)	66.7 (58.5–71.2)	61.9 (59.4–72.5)	56.7 (53.0–67.6)	68.7 (63.8–72.0)	**0.01**
Before ICI treatment Eosin (%)	1.9 (1.05–4.3)	4.8 (0.40–5.10)	4.00 (2.03–4.68)	1.6 (0.80–2.43)	0.09
Before ICI treatment Hb (g/dL)	10.8 (10.1–12)	12.7 (10.2–12.9)	11.4 (9.92–12.9)	10.8 (10.0–11.7)	0.29
Before ICI treatment Plt (×10^4^/μL)	26.4 (20.4–31.1)	20.5 (19.7–31.2)	24.1 (20.4–31.6)	27.7 (20.9–31.2)	0.66
Before ICI treatment Alb (g/dL)	3.7 (3.40–3.95)	3.7 (3.30–3.90)	3.7 (3.42–4.00)	3.6 (3.38–3.90)	0.90
Before ICI treatment CRP (mg/dL)	0.23 (0.09–0.92)	0.23 (0.09–2.84)	0.25 (0.09–0.59)	0.23 (0.08–1.04)	0.93
Before ICI treatment PNI	42.6 (39.3–45.8)	42.6 (38.8–46.2)	44.8 (39.1–50.0)	41.6 (39.7–44.1)	0.49

*Note:* Categorical variables are presented as proportions. Non‐normally distributed variables are reported as medians with interquartile ranges. Categorical data were compared using the chi‐square test or Fisher’s exact test. Non‐normally distributed data were compared using the Kruskal–Wallis test. Abbreviations: IQR, interquartile range; PNI, prognostic nutritional index.

### 3.3 Downregulation of HLA‐A/B/C and Ectopic HLA‐DR Expression in Tumor Cells Predicted a Favorable Response to ICI Therapy

**FIGURE 2:**
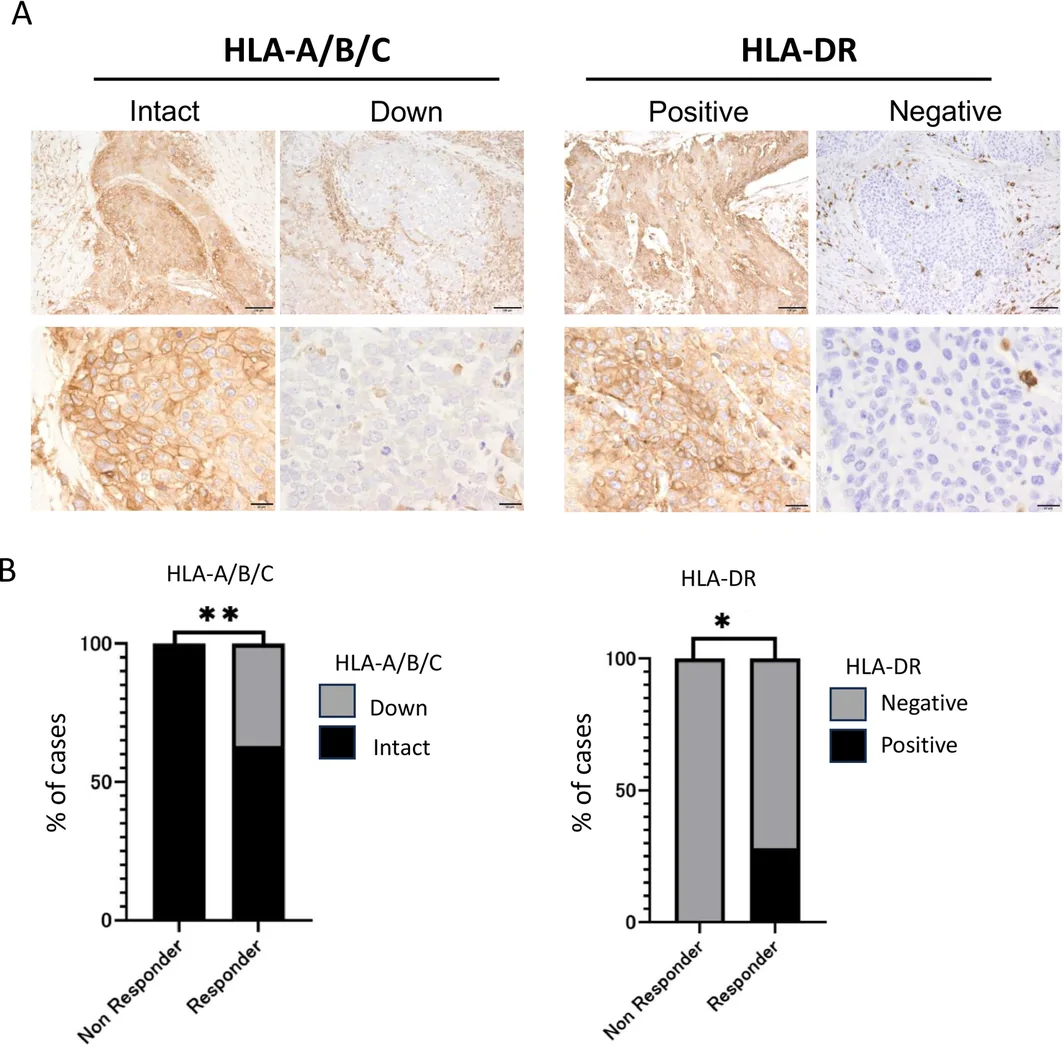
HLA‐A/B/C and HLA‐DR expression in tumor tissues. (A) Representative image of HLA‐A/B/C and HLA‐DR (upper panels; ×100, lower panels; ×400) were presented. Immune cells were positive for both antigens and assessed as internal positive control. (B) Bar graphs show the proportions of cases classified as intact or downregulated HLA‐A/B/C expression and as positive or negative HLA‐DR expression. Black bars represent intact or positive expression, and gray bars represent downregulated or negative expression. *p < 0.001, **p < 0.01.

**FIGURE 3:**
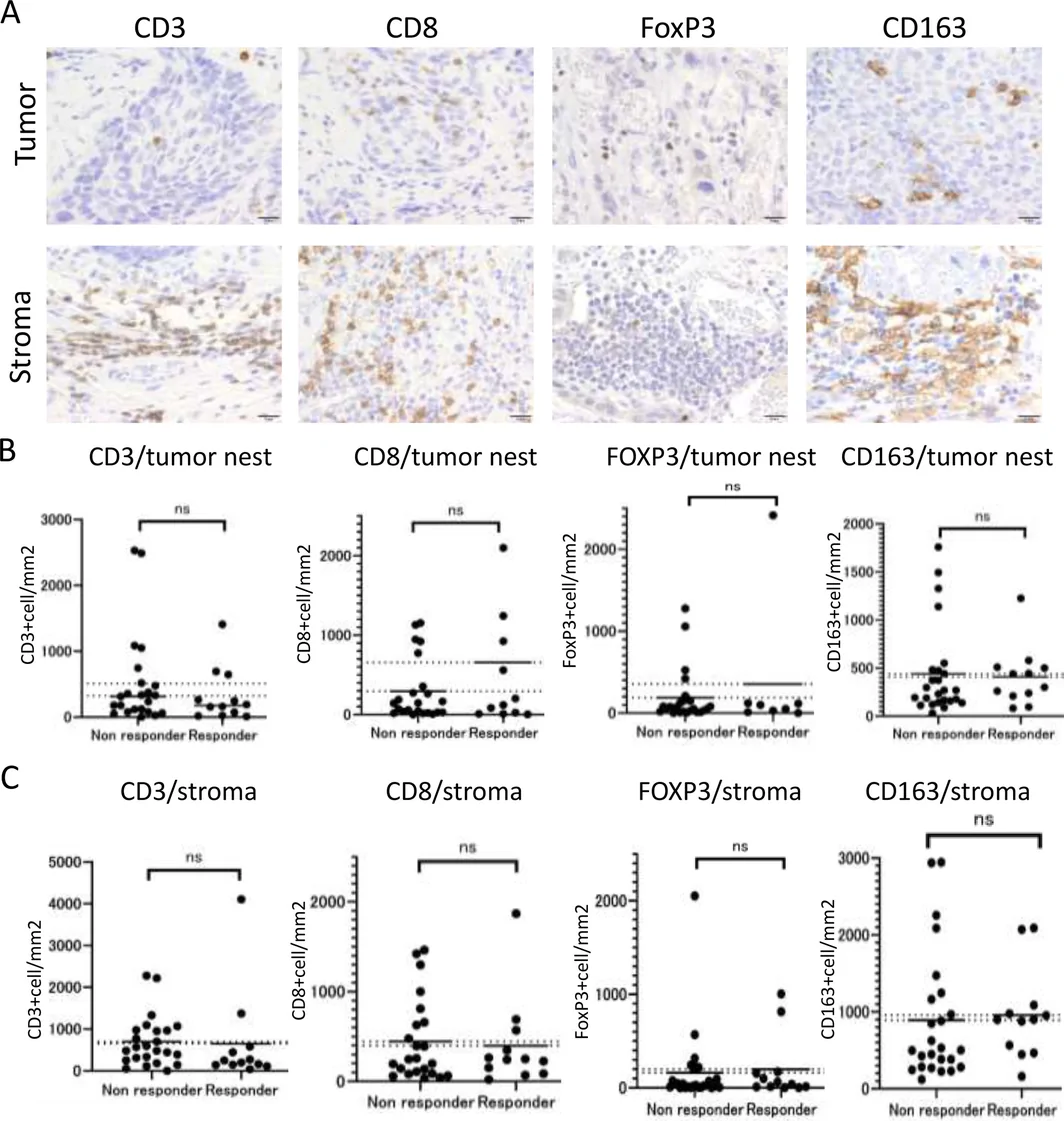
Immunohistochemical staining and quantitative analysis of tumor‐infiltrating immune cells in patients with ESCC treated with ICIs. (A) Immunohistochemistry for CD3, CD8, FoxP3, and CD163 was performed on tumor tissues. Quantitative digital image analysis of positive cells were evaluated in tumor nests (B) and tumor stromata (C). ns, not significant.

The results of an evaluation of HLA‐A/B/C antigens and HLA‐DR expression are shown in Figure 2A. Downregulation of HLA‐A/B/C was observed in 10 cases (22.2%), whereas ectopic expression of HLA‐DR was detected in 11 (24.4%). Statistical analyses demonstrated that responders exhibited both HLA‐A/B/C downregulation and ectopic HLA‐DR expression more frequently than nonresponders (Figure 2B). As summarized in Tables 3 and 4, patients with downregulated HLA‐A/B/C expression had higher median hemoglobin levels; their PNI values also tended to be higher, but this difference did not reach statistical significance. Similar trends were observed in patients with HLA‐DR–positive tumors, again without a statistically significant difference in PNI. Tumor‐infiltrating immune cells, including CD3+, CD8+, FoxP3+, and CD163+ cells, were evaluated by immunohistochemistry (Figure 3A). Quantitative analyses of cell densities in the tumor nests and stromal areas are presented in Figure 3B,C, respectively. No significant differences in immune cell densities were observed between responders and nonresponders in either compartment.

**TABLE 3:** Clinical characteristics according to HLA‐A/B/C expression.

Characteristic median (IQR)	All patients (*n* = 45)	HLA‐A/B/C expression in tumor cells		*p*‐value
		Intact (*n* = 35)	Downregulated (*n* = 10)	
Before ICI treatment WBC (×10^3^/μL)	5.3 (4.0–6.5)	4.8 (3.83–5.83)	5.6 (4.80–7.25)	0.71
Before ICI treatment Neut (%)	66.7 (58.5–71.2)	67.3 (61.8–70.9)	61.9 (58.0–74.2)	0.91
Before ICI treatment Eosin (%)	1.9 (1.05–4.3)	1.60 (0.65–2.90)	1.9 (1.45–4.70)	0.59
Before ICI treatment Hb (g/dL)	10.8 (10.1–12)	10.8 (9.88–11.9)	12.9 (11.3–13.5)	**0.02**
Before ICI treatment Plt (×10^4^/μL)	26.4 (20.4–31.1)	23.7 (19.2–30.8)	27.2 (24.0–31.9)	0.57
Before ICI treatment Alb (g/dL)	3.7 (3.40–3.95)	3.7 (3.40–3.98)	3.9 (3.55–4.25)	0.22
Before ICI treatment CRP (mg/dL)	0.23 (0.09–0.92)	0.14 (0.06–0.96)	0.31 (0.15–0.50)	0.45
Before ICI treatment PNI	42.6 (39.3–45.8)	42.7 (39.8–44.9)	46.2 (41.4–51.0)	0.13

**TABLE 4:** Clinical characteristics according to HLA‐DR expression.

Characteristic median (IQR)		HLA‐DR expression in tumor cells		*p*‐value
	All patients (*n* = 45)	Positive (*n* = 11)	Negative (*n* = 34)	
Before ICI treatment WBC (×10^3^/μL)	5.3 (4.0–6.5)	5.4 (4.00–5.60)	5.1 (3.88–6.53)	0.64
Before ICI treatment Neut (%)	66.7 (58.5–71.2)	67.9 (58.2–71.4)	66.4 (61.0–71.1)	0.93
Before ICI treatment Eosin (%)	1.9 (1.05–4.3)	0.6 (0.30–1.80)	1.8 (1.23–3.40)	0.17
Before ICI treatment Hb (g/dL)	10.8 (10.1–12)	11.2 (10.5–12.0)	10.8 (9.74–12.2)	0.60
Before ICI treatment Plt (×10^4^/μL)	26.4 (20.4–31.1)	22.4 (21.6–27.2)	27.7 (20.0–31.4)	0.72
Before ICI treatment Alb (g/dL)	3.7 (3.40–3.95)	3.6 (3.50–4.00)	3.7 (3.40–3.93)	0.78
Before ICI treatment CRP (mg/dL)	0.23 (0.09–0.92)	0.07 (0.03–0.32)	0.21 (0.09–0.89)	0.57
Before ICI treatment PNI	42.6 (39.3–45.8)	42.8 (41.2–45.4)	43.0 (39.9–46.3)	0.77

### 3.4 High PD‐L1 Expression in TAMs Was Associated With Increased Immune Responses to Tumors

**FIGURE 4:**
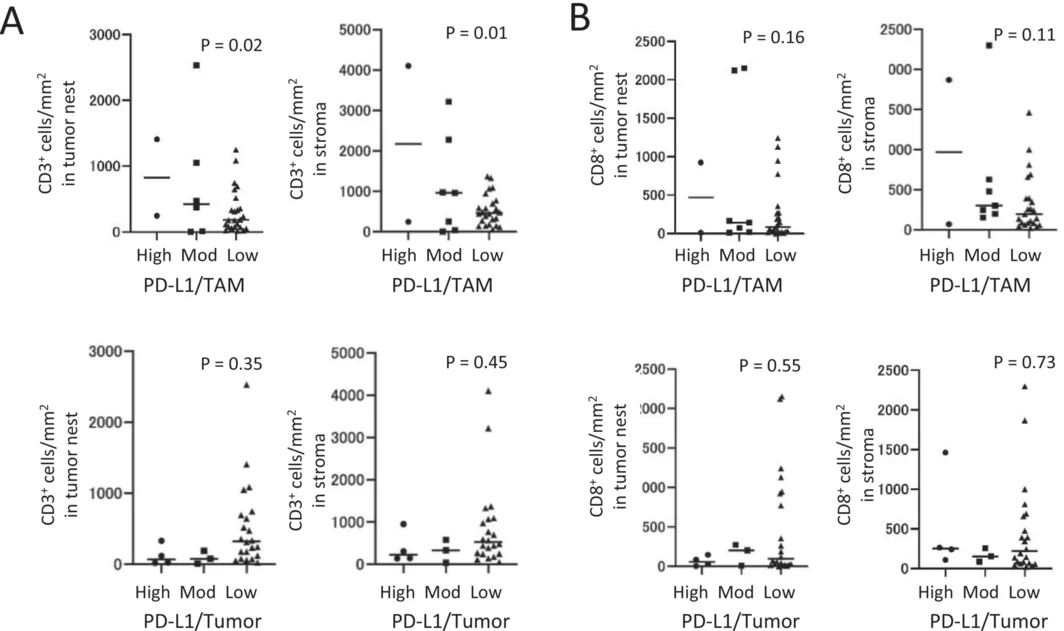
Association between PD‐L1 expression and lymphocyte infiltration. (A) The association between CD3+ lymphocytes in tumor nests and stromata and PD‐L1 expression was examined. (B) The association between CD+ lymphocytes in tumor nests and stromata and PD‐L1 expression was examined.

To explore the immunological background associated with PD‐L1 expression further, we analyzed the relationship between tumor‐infiltrating lymphocytes and PD‐L1 status. Regarding CD3+ T‐cell density, tumors with high PD‐L1 expression on TAMs showed significantly higher densities of CD3+ T cells in both the stromal area and tumor nests, whereas PD‐L1 expression in tumor cells was not associated with CD3+ T‐cell density (Figure 4A). By contrast, no significant association was observed between CD8+ T‐cell density and PD‐L1 expression (Figure 4B). Neither CD163+ TAM density nor FoxP3+ regulatory T‐cell density was associated with PD‐L1 expression (unpublished data).

**FIGURE 5:**
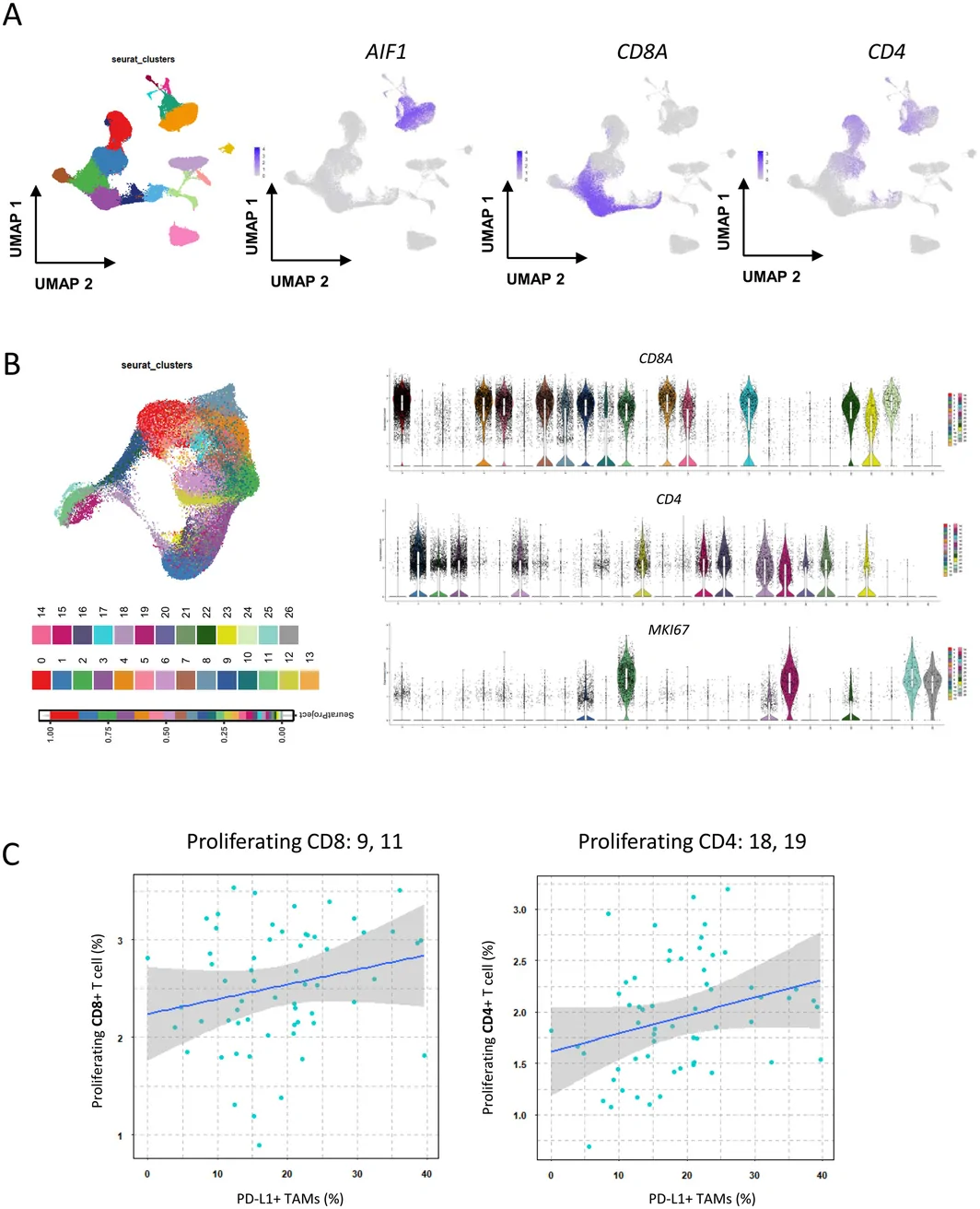
Single‐cell RNA‐sequence analysis. (A) UMAP plot of CD45+ cells from a publicly available ESCC sc‐RNA‐seq data set (GSE160269), with feature plots showing expression of AIF1 (Iba‐1), CD8A, and CD4. (B) UMAP plot (left) of extracted CD4+ and CD8+ T cells, subdivided into 27 clusters. Violin plots (right) show the expression of CD8A, CD4, and MKI67 across clusters; MKI67+ clusters were defined as proliferating T‐cell subsets. (C) Dot plots showing, for each case, the proportions of proliferating CD4+ and CD8+ T cells and CD274 (PD‐L1)–positive TAMs.

To investigate the immunological significance of PD‐L1 expression in TAMs further, we analyzed publicly available single‐cell RNA‐seq data from patients with ESCC (GSE160269), previously reported by Yang et al. [15] (Figure 5A). CD45+ immune cells were extracted, and T cells and myeloid‐lineage cells were identified based on canonical marker expression. T cells were subsequently re‐clustered, and MKI67‐expressing clusters were defined as proliferating CD8+ or CD4+ T cells (Figure 5B). Spearman's correlation analysis demonstrated a significant positive correlation between the proportion of PD‐L1+ TAMs and the proportions of proliferating CD8+ and CD4+ T cells across individual cases (Figure 5C). These findings suggest that PD‐L1 expression in TAMs is closely associated with T‐cell proliferation and may reflect an activated immune microenvironment rather than merely an immunosuppressive state.

### 3.5 Downregulation of HLA‐A/B/C Was Associated With Longer PFS

To evaluate the clinical relevance of these immune markers further, PFS was analyzed according to PD‐L1 and HLA expression status. Kaplan–Meier analyses revealed a modest but statistically significant difference in PFS according to HLA‐A/B/C status, with patients exhibiting downregulated expression demonstrating longer PFS (log‐rank *p* = 0.04). By contrast, PFS did not significantly differ according to PD‐L1 expression in TAMs (*p* = 0.85) or to HLA‐DR expression in cancer cells (*p* = 0.89) (Figure S1).

## 4 Discussion

In this study, higher PNI values were significantly associated with a favorable response to ICI therapy, whereas CRP levels showed only a nonsignificant trend toward lower values in responders. It is well established that systemic host factors, including nutritional and inflammatory status, are closely linked to treatment outcomes [16]. The PNI has repeatedly been validated as a prognostic indicator in esophageal cancer, including in patients treated with nivolumab [17]. As a composite index integrating serum albumin and lymphocyte count, the PNI reflects the interplay between nutritional reserve and systemic inflammation, both of which influence the host immune system's capacity to mount effective antitumor responses under checkpoint inhibition. Elevated CRP levels have been associated with resistance to ICI therapy across multiple cancer types [18], and their clinical significance in patients with ESCC receiving ICIs has also been reported [19]. However, conflicting results have been described in ESCC [20, 21], suggesting that CRP alone may be insufficient to predict ICI efficacy reliably in this disease. In the present cohort, although CRP tended to be lower in responders, the difference did not reach statistical significance. Higher hemoglobin levels and PNI values were significantly associated with an improved response to ICI therapy. These findings underscore the potential importance of the systemic host condition in determining immunotherapeutic efficacy. Hemoglobin levels and the PNI are recognized indicators of nutritional status, inflammatory burden, and immune competence, and cancer‐related anemia and systemic inflammation are frequently linked to immunosuppressive states and impaired T‐cell function [16, 17, 18, 22].

Although we observed associations between systemic hematologic parameters and immune‐related tumor markers, these findings should not be interpreted as evidence of direct mechanistic interactions between hemoglobin or the PNI and PD‐L1 or HLA expression. Rather, they suggest that systemic physiological status may influence, or coexist with, tumor immune characteristics that modulate the responsiveness to immunotherapy. Clinically, these results indicate that readily obtainable hematologic markers may serve as complementary indicators when considering ICI treatment strategies. Nevertheless, prospective validation in larger cohorts is warranted. PD‐L1 expression is a central immune evasion mechanism, and PD‐L1 immunohistochemistry is widely used as a companion diagnostic in several cancers [23]. In ESCC, the combined positive score, which incorporates PD‐L1 expression on both tumor and immune cells, is the most commonly used biomarker in clinical practice and trials, including KEYNOTE‐590 [3]. Consistent with this background, increased PD‐L1 expression was associated with a favorable response to ICI therapy in the present study. We evaluated PD‐L1 expression separately in TAMs and cancer cells. PD‐L1 expression in TAMs and cancer cells was linked to a favorable response. Multiple studies have clarified mechanisms regulating PD‐L1 in cancer cells, indicating that PD‐L1 overexpression can be driven by genetic alterations and inflammatory cytokines such as interferons [24]. In macrophages, PD‐L1 expression is preferentially induced by interferons, and lymphocyte‐derived GM‐CSF can further enhance PD‐L1 upregulation [25]. In our cohort, higher PD‐L1 expression in TAMs was correlated with increased CD3+ T‐cell infiltration in the stroma, and sc‐RNA‐seq analysis revealed a significant positive correlation with T‐cell proliferation. Because T‐cell proliferation reflects T‐cell activation, these findings suggest that factors derived from activated T cells may promote PD‐L1 expression in TAMs.

Within the tumor immune microenvironment, our analyses showed that the absolute densities of CD3+, CD8+, FoxP3+, and CD163+ cells did not differ significantly between responders and nonresponders. This suggests that quantitative immune cell infiltration alone may be insufficient as a predictive marker. Accumulating evidence indicates that the spatial distribution and functional orientation of immune cells—rather than their sheer abundance—are critical determinants of whether tumors are “hot” or “cold.” For example, CD8+ T cells located near antigen‐presenting cells or within tertiary lymphoid structures may have greater predictive value than bulk cell counts. This concept aligns with emerging views that immune contexture and topology are essential for predicting the immunotherapy response in ESCC [3, 4].

Our antigen‐presentation analyses yielded paradoxical but intriguing results. Responders more frequently displayed low HLA class I expression accompanied by high HLA class II expression. Ordinarily, downregulation of HLA‐I is considered a mechanism of immune escape, enabling tumor cells to evade CD8+ T‐cell recognition [11]. Indeed, HLA‐I defects and β2‐microglobulin loss are well‐established causes of resistance to immunotherapy. However, in our cohort, HLA‐I downregulation was enriched among responders. This pattern may indicate that HLA‐II‐mediated CD4+ T‐cell activation plays a more dominant role than HLA‐I in shaping the therapeutic response. Studies in melanoma and other cancers have shown that HLA‐II expression predicts improved outcomes following PD‐1/PD‐L1 blockade [26, 27]. Furthermore, reduced HLA‐I expression may sensitize tumor cells to natural killer (NK) cell‐mediated “missing‐self” recognition, providing an alternative cytotoxic pathway that could synergize with checkpoint inhibition [11]. Together, these observations suggest that ESCC responses to ICIs may depend on a cooperative axis involving CD4+ T, NK, and professional antigen‐presenting cells, rather than relying solely on classical CD8+ T‐cell recognition. Notably, the combined immunophenotypic profile of HLA‐A/B/C downregulation and HLA‐DR positivity may represent a distinct tumor immune contexture associated with improved responsiveness to ICI therapy. While HLA class I downregulation is conventionally viewed as a mechanism of immune escape, its coexistence with HLA class II expression may reflect a shift toward CD4+ T‐cell‐mediated immune activation. From a clinical perspective, integrating HLA class I and class II expression patterns may allow more refined patient stratification than assessment of either marker alone. However, due to the limited sample size, a formal evaluation of the interaction or synergistic effects between these markers was not feasible, and validation in larger cohorts will be necessary.

Using sc‐RNA‐seq, Yang et al. [28] recently demonstrated that increased numbers of SPP1+ TAMs contribute to resistance to immunochemotherapy in ESCC through CD44–SPP1 interactions. SPP1, also known as osteopontin, is a well‐characterized oncogenic factor secreted by cancer and immune cells. It has been highlighted as a marker of monocyte‐derived TAMs [29] and implicated in cancer progression in lung and colorectal cancers [30, 31]. Because PD‐L1+ TAMs were associated with better clinical responses in the present study, we hypothesized a negative relationship between PD‐L1+ and SPP1+ TAM populations. In addition, sc‐RNA‐seq showed that SPP1+ and PD‐L1+ TAM clusters localized to distinct regions on UMAP (Figure S2), although no quantitative correlation was observed between the numbers of SPP1+ and PD‐L1+ TAMs (Figure S2). Therefore, in addition to PD‐L1, SPP1+ TAMs may serve as a useful biomarker for predicting ICI efficacy.

This study has several limitations. First, this was a retrospective analysis conducted at a single institution with a relatively small sample size, which may limit the generalizability of the findings. Second, ICI regimens were heterogeneous and included both monotherapy and combination therapy administered at different lines of treatment, reflecting real‐world clinical practice. Although this enhances clinical relevance, the potential influence of treatment regimen on therapeutic response should be considered when interpreting the present findings. Third, due to the limited number of events, comprehensive multivariable analyses adjusting for all potential confounders were not feasible. In addition, CD4 immunohistochemistry was not performed in this study. Quantitative assessment of CD4+ T‐cell infiltration may be confounded by CD4 staining in non–T‐cell populations, such as macrophages. Furthermore, CD3+ and CD8+ T‐cell densities were quantified independently using three randomly selected 1‐mm^2^ regions at the tumor invasive front; therefore, these markers were not evaluated in identical microscopic fields, precluding a reliable estimation of a CD3 + CD8– proxy for CD4+ T‐cell infiltration. Future studies incorporating dedicated CD4+ T‐cell analyses are needed to clarify the role of HLA class II‐mediated immune responses in ESCC. Finally, external validation in an independent cohort is warranted to confirm the predictive value of the identified immune markers.

Collectively, our data indicate that ICI responses in ESCC are governed by a multilayered network involving systemic immune fitness, PD‐L1 expression on immune cells, and the balance of antigen‐presentation pathways. In addition to PD‐L1 immunohistochemistry, HLA expression should be evaluated alongside PD‐L1 by immunohistochemistry. Given that PD‐L1 expression in TAMs correlated strongly with T‐cell proliferation, TAM PD‐L1 may serve as a useful surrogate marker of T‐cell activation/proliferation within the tumor immune microenvironment.

## Author Contributions

**Yukio Fujiwara:** software, formal analysis. **Yoshifumi Baba:** conceptualization, software, data curation, supervision. **Kazuto Harada:** data curation. **Cheng Pan:** software, formal analysis.

## Funding

The authors have nothing to report.

## Ethics Statement

Approval of the research protocol: The protocol of this study was approved by the human ethics review committee of the Graduate School of Medicine, Kumamoto University, and carried out according to the Declaration of Helsinki and Good Clinical Practice Guidelines.

## Consent

The authors have nothing to report.

## Conflicts of Interest

The authors declare no conflicts of interest.

## Supporting information


**Figure S1:** Association between immune markers and progression‐free survival time. Kaplan–Meier analysis was performed to test the correlation between PD‐L1 and HLA expression status in progression‐free survival (PFS). P‐values were evaluated using the log‐rank test.
**Figure S2:** Association between PD‐L1+ and SPP1+ TAMs in esophageal cancer. Single‐cell RNA‐sequence data from Figure 5 were reanalyzed. UMAP plot of the TAMs and their expression of SPP1 and CD274 (PD‐L1).

## Data Availability

The data that support the findings of this study are available on request from the corresponding author. The data are not publicly available due to privacy or ethical restrictions.
