# Supplementary material for: Clinical Significance of PD‐L1 and HLA Expression in Esophageal Squamous Cell Carcinoma in Response to Immunotherapy
Source: Ann Gastroenterol Surg. 2026 May 8:10.1002/ags3.70227. Online ahead of print. doi: 10.1002/ags3.70227 (PMC13394168; doi:10.1002/ags3.70227)
Supplement: Supplementary file 1 — Figure S1: Association between immune markers and progression‐free survival time. Kaplan–Meier analysis was performed to test the correlation between PD‐L1 and HLA expression status in progression‐free survival (PFS). P‐values were evaluated using the log‐rank test. Figure S2: Association between PD‐L1+ and SPP1+ TAMs in esophageal cancer. Single‐cell RNA‐sequence data from Figure 5 were reanalyzed. UMAP plot of the TAMs and their expression of SPP1 and CD274 (PD‐L1). [file AGS3-9999-0-s001.pptx]

## Slide 1
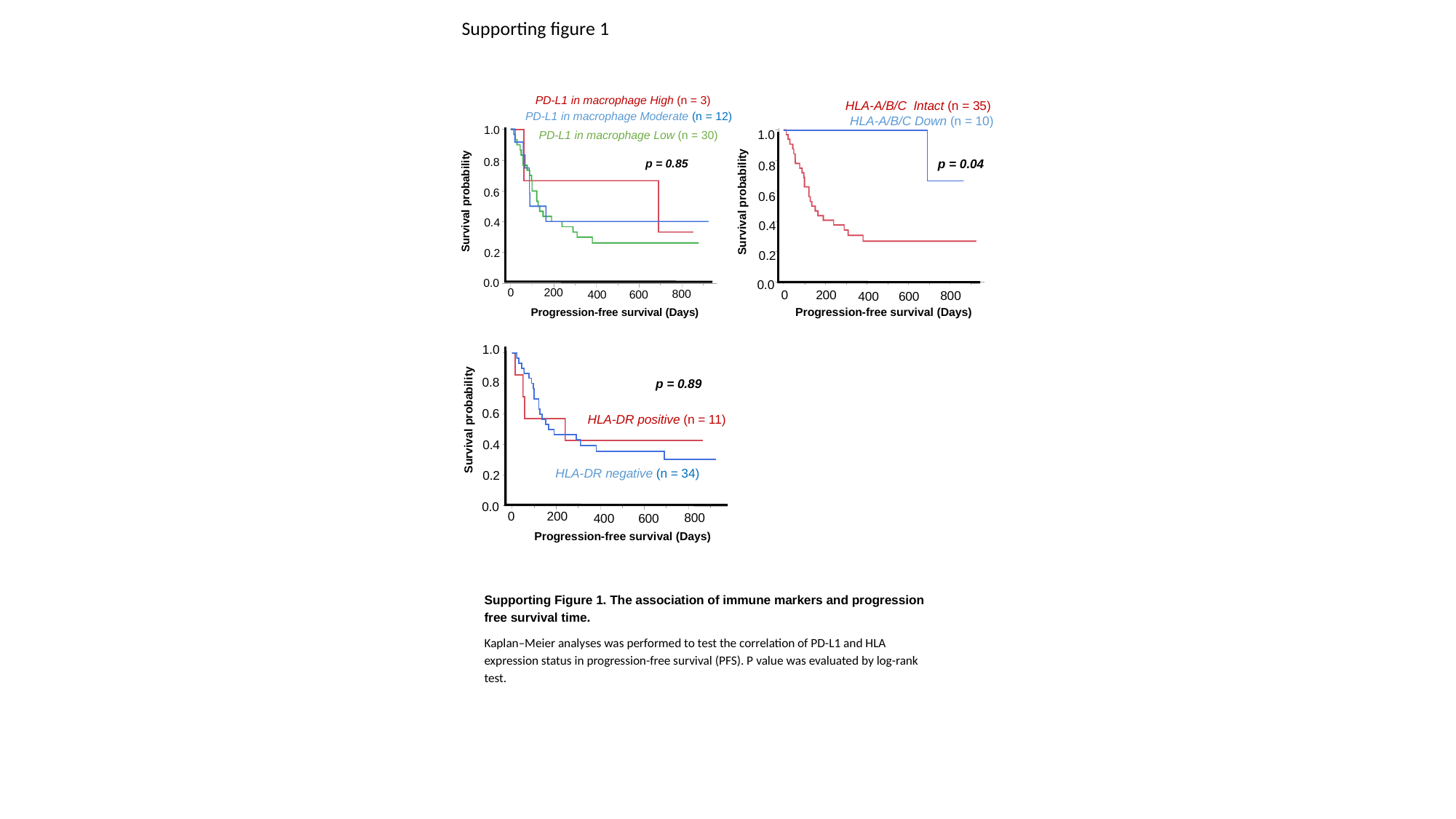

Supporting figure 1
HLA-A/B/C Intact (n = 35)
HLA-A/B/C Down (n = 10)
1.0
Survival probability
p = 0.04
0.8
0.6
0.4
0.2
0.0
0
200
800
400
600
Progression-free survival (Days)
PD-L1 in macrophage High (n = 3)
PD-L1 in macrophage Moderate (n = 12)
PD-L1 in macrophage Low (n = 30)
1.0
Survival probability
p = 0.85
0.8
0.6
0.4
0.2
0.0
0
200
800
400
600
Progression-free survival (Days)
1.0
Survival probability
p = 0.89
0.8
0.6
HLA-DR positive (n = 11)
0.4
HLA-DR negative (n = 34)
0.2
0.0
0
200
800
400
600
Progression-free survival (Days)
Supporting Figure 1. The association of immune markers and progression free survival time.
Kaplan–Meier analyses was performed to test the correlation of PD-L1 and HLA expression status in progression-free survival (PFS). P value was evaluated by log-rank test.

## Slide 2
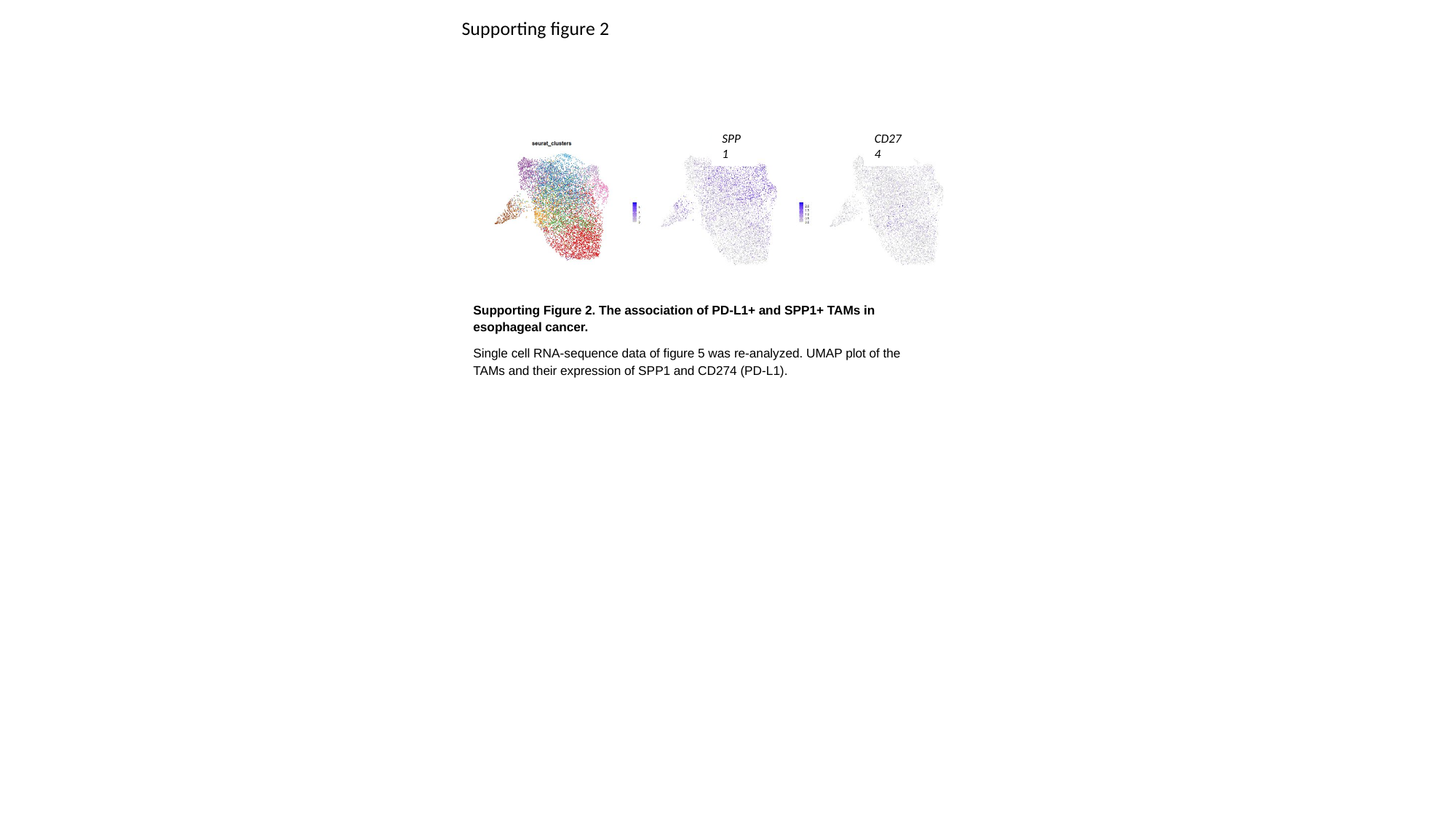

Supporting figure 2
SPP1
CD274
Supporting Figure 2. The association of PD-L1+ and SPP1+ TAMs in esophageal cancer.
Single cell RNA-sequence data of figure 5 was re-analyzed. UMAP plot of the TAMs and their expression of SPP1 and CD274 (PD-L1).
